# Peritumoral Invasion and Survival in Patients with Oral Squamous Cell Carcinoma—The Role of Perineural and Lymphovascular Invasion

**DOI:** 10.3390/cancers17172812

**Published:** 2025-08-28

**Authors:** Samer George Hakim, Ubai Alsharif, Mohamed Falougy, Lars Tharun, Dirk Rades, Christiane Kümpers, Justus Jensen

**Affiliations:** 1 Department of Oral and Maxillofacial Surgery, Head and Neck Cancer Center, University Hospital Schleswig-Holstein, Campus Lübeck, 23562 Lübeck, Germany; samer.hakim@uni-luebeck.de (S.G.H.); mohamed.falougy@uksh.de (M.F.); 2 Department of Oral and Maxillofacial Surgery, Helios Medical Center, 19055 Schwerin, Germany; 3 Department of Oral and Maxillofacial Surgery, Dortmund General Hospital, 44145 Dortmund, Germany; ubai.alsharif@uni-wh.de; 4 Faculty of Health, Witten/Herdecke University, 58455 Witten, Germany; 5 Department of Pathology, University Hospital Schleswig-Holstein, Campus Lübeck, 23562 Lübeck, Germanychristiane.kuempers@uksh.de (C.K.); 6 Department of Radiation Oncology, University Hospital Schleswig-Holstein, Campus Lübeck, 23562 Lübeck, Germany; dirk.rades@uksh.de

**Keywords:** oral squamous cell carcinoma, overall survival, locoregional recurrence-free survival, lymphatic invasion, perineural invasion, vascular invasion, lymphovascular invasion

## Abstract

**Background/Objectives:** Perineural (PnI), lymphatic (LI), and vascular invasion (VI) in tumor specimens are supposed to worsen the clinical course of oral squamous cell carcinoma (OSCC) and negatively influence survival outcomes. Despite this, these histologic features have not been implemented in the international staging recommendation for OSCC and their prognostic role remains questionable due to inconsistent findings in the related literature. **Methods:** To investigate the impact of PnI, LI, and VI on oral cancer-specific (OCSS), recurrence-free (RFS), and overall survival (OS), we hypothesized that these histologic features are independent risk factors for poor survival and therefore considered within a prospectively maintained single-center cohort of patients with OSCC. LI and VI were assessed together and reported as lymphovascular invasion (LVI). **Results:** This study included 439 patients with primary OSCC. Sixty-nine Patients (21.9%) had at least one of the two risk factors. Within the 5-year follow-up period, 61 of these patients (64%) died, and 30 patients (31%) developed locoregional recurrences. Both perineural and lymphovascular invasion were strongly correlated with the presence of lymph node metastasis. PnI and LVI were investigated separately using an adjusted Cox’s proportional hazards regression model. In addition to higher tumor size and the presence of nodal disease (higher stage) the presence of LVI was associated with poor OS, OCSS, and RFS on multivariate analysis, while PnI was associated with reduced OS. In stage III/IV postoperative radiotherapy improved survival in patients with PnI but not with LVI. **Conclusions:** We conclude that the evidence of LVI in tumor specimens should be considered a high-risk factor when planning adjuvant treatment and monitoring patients with OSCC.

## Simple Summary

Oral squamous cell carcinoma continues to represent a major health burden, particularly due to its poor prognosis in advanced stages. Accurate identification of histopathologic features that influence tumor progression and survival is crucial for improving staging systems and guiding therapy. Perineural invasion, defined as the spread of tumor cells along nerves, and lymphovascular invasion, defined as tumor spread into blood or lymphatic vessels, have been proposed as important prognostic factors. However, their impact on disease course and treatment decisions remains uncertain. In this study, we analyzed a single-center cohort to investigate the relationship of these features with regional metastasis, recurrence, and survival. We also evaluated whether additional postoperative adjuvant therapy may improve outcomes in affected patients. Our findings suggest that both perineural and lymphovascular invasion are associated with poorer prognosis, with lymphovascular invasion emerging as an independent predictor, supporting its inclusion in future staging and treatment recommendations.

## 1. Introduction

Despite the stable age-standardized incidence rate for decades, mortality of oral squamous cell carcinoma (OSCC) still represents an increasingly important health problem [1,2]. Its relatively poor prognosis in advanced stages (stage III–IV) has drawn attention to the early diagnosis and treatment based on emerging histological and immunohistological risk factors. These factors affect the biological behavior of the tumor and, subsequently, the course of the disease. These risk factors are increasingly influencing the current TNM classification and the recommendation of treatment of OSCC. The focus has been turned to the role of inherent histopathologic features of the tumor, such as depth of invasion (DOI), HPV-positivity, tumor budding [3], perineural (PnI), and lymphovascular invasion (LVI) as well as their role in invasion and metastasis [4,5,6,7,8].

While the role of perineural invasion in locoregional recurrence has been investigated thoroughly [9,10,11,12,13], the prognostic role of lymphovascular invasion (LVI) is relatively understudied. LVI is defined as the presence of tumor cells within defined endothelial-lined spaces, either lymphatic or blood vessels, detected by hematoxylin and eosin (H&E) staining alone and/or supported by immunohistochemistry [14]. In a recent review, the prevalence of LVI in OSCC ranged from 3% to 90%, showing a wide variation field [14]. This high discrepancy raises concerns about the reliability of this histologic feature as a prognostic marker. Extremely low or high prevalence limits the ability to assess its significance and complicates the interpretation of its validity as a potential risk factor for survival outcomes.

Theoretically, three explanations for these divergent results can be postulated: first, the different diagnostic standards for the definition of the LVI [15,16]; second, the merging of the two histopathologic features, lymphatic invasion (LI) and vascular invasion (VI), into one variable, LVI; and third, the presence of further relevant associated risk factors, which were not considered adequately in the multivariate analysis.

In view of the correlation of LVI with the survival outcomes of patients with OSCC, the vast majority of the previous studies either investigated selected cases from pathologic records, failed to adjust for competing risk factors and confounders. Many of these studies also analyzed only the overall survival instead of the much more relevant cancer-specific and recurrence-free survivals [8,17,18].

With its postulated prognostic value, the role of PnI in current TNM staging systems remains limited. Its importance for stratification and treatment guidelines is hampered by the heterogeneous diagnostic criteria for its evidence, which range from plain evidence to multifocal and extratumoral dissemination as well as dependence from tumor site. Therefore, the current related literature remains divided regarding the benefit of treatment escalation by postoperative radiotherapy, resulting in wide heterogeneity in the treatment protocols [13,19,20,21]. In clinical routine, the evidence of PnI in tumor specimens generally encourages the recommendation for radiotherapy, regardless of T or N status. However, LVI still seen controversially in this concern and treatment guidelines do not necessarily require therapy escalation, unless other criteria are fulfilled (e.g., T3–4 and N+). This strategy may worsen the survival outcome in these patients and lead to biasing the weighting of confounders in the survival analysis, if the impact of LVI on it is overseen.

Further, PnI, LI, and VI were frequently reported to be partially correlated, though, most studies did not control for all three variables simultaneously when estimating the survival outcomes. If PnI and LVI contribute to poor prognosis, is there any role of therapy escalation in improving the survival outcome in Patients with OSCC?

To explore the three potential histologic risk factors in view of their impact on cancer-specific, recurrence-free, and overall survival, we hypothesized that LVI is an independent risk factor and considered it separately along with the PnI within a prospectively maintained single-center cohort. The aims of the present study were as follows:

1. To identify the factors associated with PnI and LVI.
2. To assess the association of PnI and LVI with regional neck metastasis.
3. To evaluate the association of PnI and LVI with survival outcomes.
4. To investigate the effect of postoperative radiotherapy in the different subgroups of patients.

## 2. Methods

### 2.1. Study Population and Data Acquisition

**Figure 1:**
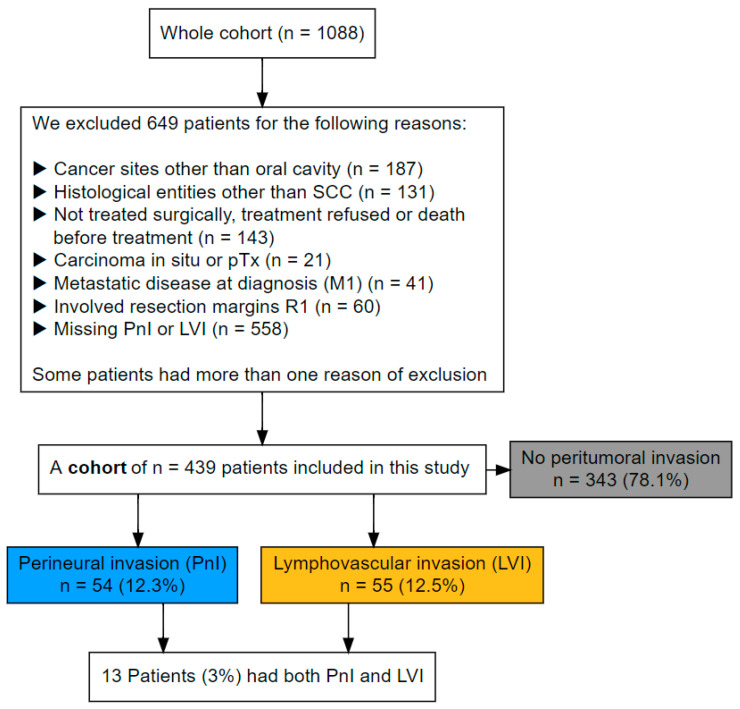
Flowchart of patients’ selection/exclusion and number of included patients by group stratified for PnI and LVI.

All patients with a primary OSCC from a cohort of 1088 patients were identified within the tumor data bank of the Department of Maxillofacial Surgery of the University Medical Centre of Lübeck, Germany. All patients underwent primary diagnostic investigation and therapy between 1992 and 2019. For this study, we only included curatively treated patients who had known status of peritumoral infiltration. Patients with incomplete tumor resection (R1-status), distant metastasis, cancer of unknown primary, and a history of head and neck cancer with or without irradiation were excluded. Figure 1 shows the criteria for exclusion.

Patients’ data were obtained from a prospectively maintained single-center cohort and included demographics, risk factors, and clinical tumor characteristics. Treatment decisions were available at the baseline and at each follow-up. We used the updated version of Charlson’s comorbidity index (CCI) to capture comorbidities and categorized CCI into no significant comorbidities (score = 0) and at least one score point (CCI score ≥ 1) [22,23].

### 2.2. Histopathology

For histopathological evaluation, the specimens were first assessed macroscopically, and, as a rule, the tumor was entirely embedded in relation to the adjacent tissue and the resection margins. All paraffin-embedded tumor blocks were sectioned at a thickness of 4 μm and stained with haematoxylin and eosin. Histopathological evaluation was performed by two experienced specialists in pathology. All sections of the tumors were studied histologically for perineural invasion (PnI), lymphatic invasion (LI), and vascular invasion (VI). This was usually performed purely based on HE morphology, as is standard practice in routine diagnostics.

**Figure 2:**
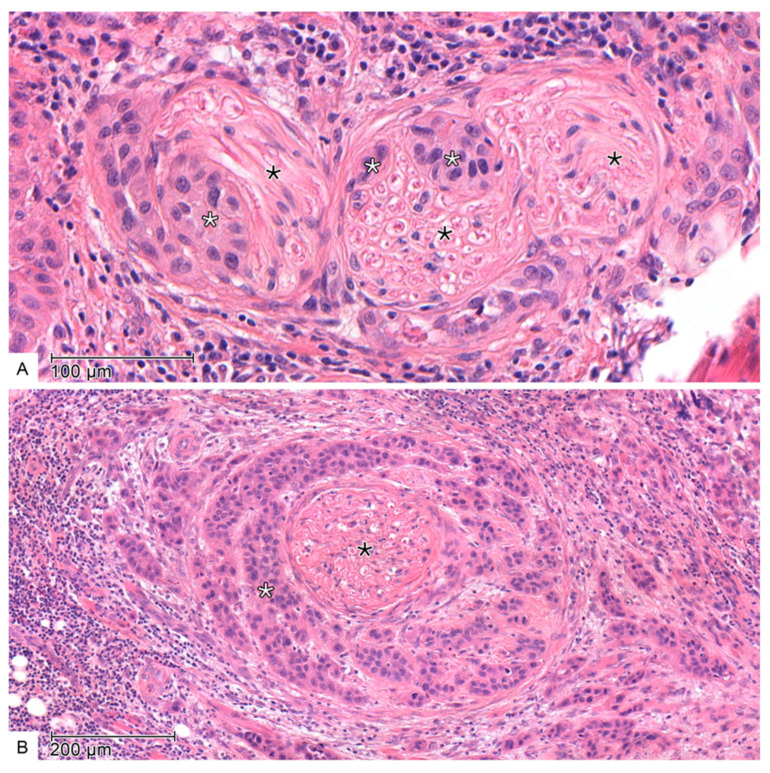
Microscopic images with evidence of perineural invasion in haematoxylin-eosin staining—(**A**): Infiltration of the endoneural space of a peripheral nerve (black star) by tumor cells (white star). (**B**): The tumor cells (white star) adhere closely to the peripheral nerve (black star) and encircle it completely.

PnI, as shown in Figure 2, was defined as tumor cell infiltration in any layer of the nerve sheath or tumor involving more than one-third of the nerve circumference [24,25]. Additional immunohistochemistry with S100 was not performed.

**Figure 3:**
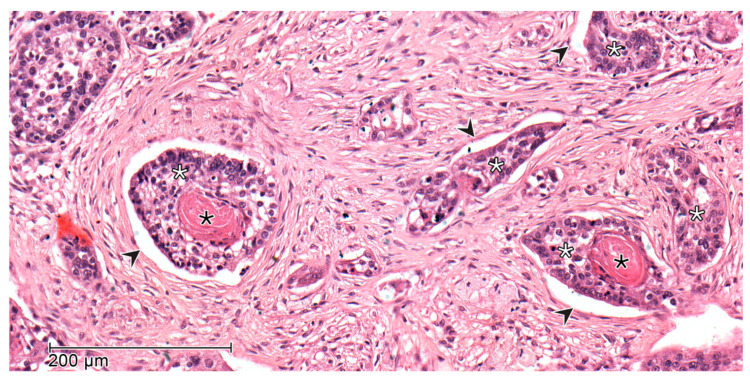
Microscopic image with evidence of lymphatic invasion in hematoxylin-eosin staining—Tumor cells of a squamous cell carcinoma (white star) with the formation of pathognomonic keratin pearls (black star) within endothelium lined lymph vessels (black arrow).

**Figure 4:**
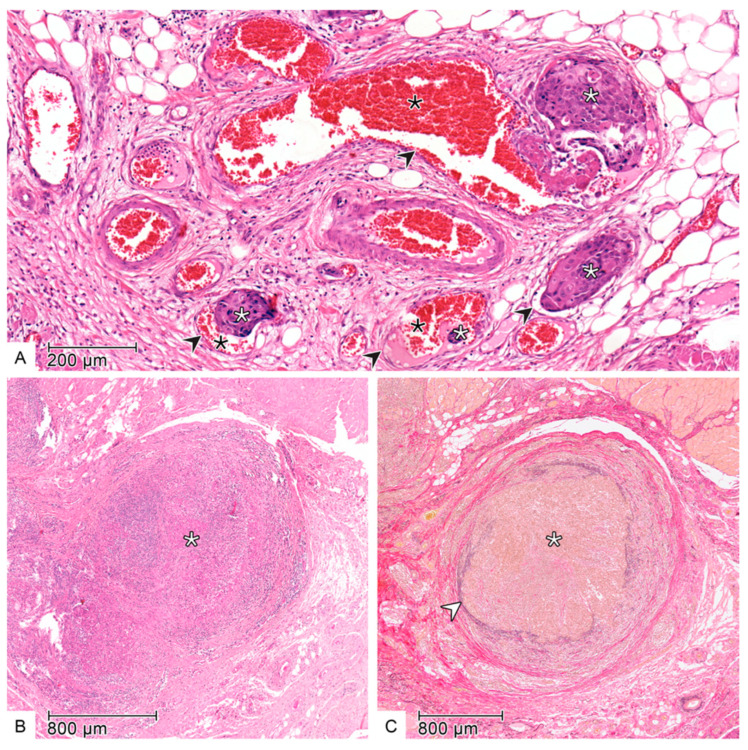
Microscopic images with evidence of vascular invasion in haematoxylin-eosin staining (**A**,**B**) and Elastica-van Gieson staining (**C**—**A**): Tumor cells of a squamous cell carcinoma (white star) located within endothelium lined blood vessels (black arrow). The vessels are clearly identifiable by their lumina filled with erythrocytes (black star). (**B**): Tumor cell cluster (white star) with suspected vascular invasion. The vessel lumen is completely obstructed by the tumor cells, and the vessel wall is challenging to delineate. (**C**): The same tissue section as in (**B**). The EVG staining stains the elastic fibers of the vessel wall black-violet (white arrow) and the cytoplasm of the tumor cells yellow (white star). This confirms the vascular invasion suspected in the HE-stained section (**B**).

LI and VI (Figure 3 and Figure 4) were assessed when aggregates of tumor cells were detected within or directly adjacent to the endothelial cell lining with no underlying muscular walls and invasion of the vessel layers, respectively. In most cases, HE-staining was used alone. However, in uncertain cases such as a wavy stroma surrounding tumor cells, additional Elastica van Gieson (EVG) staining was performed to confirm the presence of VI [16,26,27].

### 2.3. Follow-Up and Survival Endpoints

All patients were enrolled in a strictly controlled recall system to ensure regular follow-up within the five-year post-therapeutic period (every three months in the first two years and every six months after that). Data were available at the beginning of the study and each follow-up. Demographic data, risk factors, clinical tumor characteristics, and treatment decisions were prospectively collected. This follow-up included clinical, sonographic, and radiologic assessment and ended when patients either fulfilled 5 years of complete disease-free follow-up, decided to drop out from the regular aftercare, died, or the follow-up endpoint on the 31 March 2024 was reached. Patients who were alive at the end of follow-up were censored. All survival durations were measured from the time point of the initial diagnosis. The endpoint of overall survival (OS) was death from any cause; the endpoint of oral cancer-specific survival (OCSS) was death from oral cancer; the endpoint of locoregional recurrence-free survival (LRRFS) was the local or neck recurrence; the endpoint of local recurrence free survival (LRFS) was local recurrence only, while the endpoint of distant metastasis free survival (DMFS) was the presence of distant metastasis.

### 2.4. Statistical Analysis

The baseline data was tabulated after calculating medians and interquartile range (IQR) for skewed variables and presented stratified by PnI or LVI. The median, 2-, and 5-year survival and event probabilities, as well as 95% corresponding confidence intervals (CI), were estimated for OS using the Kaplan–Meier method and using cumulative incidence for OCSS, and LRRFS, LRFS and DMFS.

**Figure 5:**
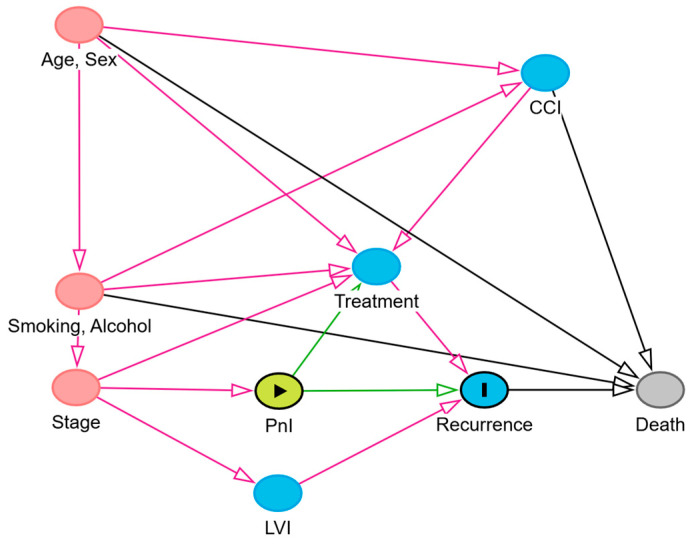
Directed acyclic graph illustrating the causal relationship between PnI (exposure) and oral cancer recurrence (outcome). PnI as the exposure is shown in green; the outcome and its ancestors are shown in blue; ancestors of both exposure and outcome are colored red; all other variables are displayed in grey.

First, to assess the prognostic factors associated with the presence of PnI and LVI, we used two multivariate bionomial regression models, for PnI and LVI, respectively. Second, to examine the association of PnI and LVI with the presence of cervical metastasis, we used several univariate binomial regression models to identifiy potential risk factors associated with cervical metastasis. Then, we eliminated those risk factors with a *p*-value less than 0.15 and introduced the remaining variables to a multivariable logistic regression model with cervical metastasis as an outcome. Third, we examined the direct effect of PnI and LVI on overall survival and the total effect of PnI and LVI on all other factors. We estimated hazards ratios (HR) and their corresponding 95% confidence intervals (CI) of the association of PnI and LVI with OS, OCSS, LRRFS, and LRFS using adjusted Cox’s proportional hazards regression models. The models for OCSS, LRRFS, LRFS, and DMFS were extended Cox’s models that accounted for competing survival events such as death from factors other than oral cancer. We identified the minimally sufficient adjustment set for survival outcomes (i.e., the smallest set of covariates required to obtain an unbiased effect estimate) using directed acyclic graph models, as shown in Figure 5. When evaluating the total effect of PnI on recurrence, tumor stage emerged as the minimal sufficient adjustment variable. Reversing the roles of PnI and LVI yielded the same result, with tumor stage alone constituting the minimal sufficient adjustment set. For completeness, we additionally included gender, smoking, and alcohol consumption in the models. These variables did not alter the effect estimates. Finally, to explore changes in effect estimates in different subgroups, we ran the same Cox’s models using the same adjustment covariates in groups stratifying by tumor stage and irradiation status. The results were presented and tabulated in a forest plot. All statistical analyses were performed using R Statistical Software (version 4.5.0; R Foundation for Statistical Computing, Vienna, Austria).

### 2.5. Ethics

On admission, all participants signed consent forms allowing their data to be collected and used anonymously for academic research. The ethics review committee of the University of Lübeck approved the study (ID 16-272A).

## 3. Results

### 3.1. Patients’ Characteristics

Out of 1088 patients presented between 1992 and 2019, 439 patients had known PnI-, LI- and VI- status and fulfilled the inclusion criteria. Ninety-six patients (21.9%) showed at least one pattern of peri-tumoral invasion in their specimens (PnI or LVI), while 343 patients (78%) had no signs of a peri-tumoral invasion.

Forty-five patients (47%) with evident PnI and/or LVI were treated by surgery alone, while 51 patients (53%) received adjuvant radio (chemo)therapy (RCT). 

In total, specimens of 54 patients (12%) showed perineural invasion (PnI+), 44 patients (10%) had lymphatic invasion (LI+), and a minority of 17 patients (3.9%) displayed vascular invasion (VI+).

As shown in Table 1, perineural invasion was observed in 12% of patients (n = 54). Compared to PnI-negative cases, PnI-positive patients exhibited more aggressive tumor characteristics. A higher proportion of T4 tumors was noted (24% vs. 16%), and UICC stage IV disease was more prevalent (46% vs. 28%). Poor tumor differentiation (24% vs. 19%) and a greater frequency of close margin resection (R0cm) or high-risk resection margin (R0hr) were also observed among PnI-positive individuals.

LVI was identified in 13% of patients (n = 55), who similarly showed more advanced disease features. The prevalence of T4 tumors was markedly higher in the LVI-positive group (36% vs. 14%), as was the occurrence of nodal metastases (N2c/3: 25% vs. 4.7%). Poor differentiation was more common (38% vs. 17%), and a substantially higher proportion of LVI-positive patients presented with UICC stage IV disease (67% vs. 25%). Tumors in this group were more frequently located in the floor of the mouth (47% vs. 32%), and high-risk resection margins (R0hr) were more frequently reported (51% vs. 27%).

### 3.2. Survival Outcomes

The median OS duration among the living patients was 59 months. In a cumulative follow-up duration of 1025 person-years, 122 patients died, resulting in a death rate of 11.9 deaths per 100 person-years.

**Figure 6:**
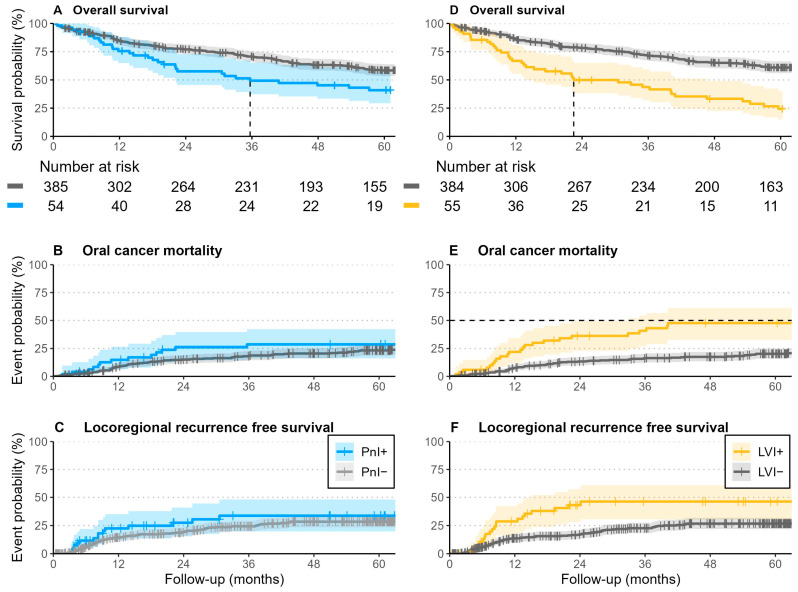
Kaplan–Meier and the cumulative incidence plots for overall, oral cancer mortality, and locoregional recurrence free survival show poor overall survival in patients with evident peritumoral invasion (PnI or LVI), whereas only LVI has a negative impact on cancer specific survival and locoregional recurrence free survival.

After 5 years of follow-up, 66% of patients were alive, whereas 20% died due to oral cancer (35% of them with evidence of PnI or LVI), and the remaining died from other causes. Within the follow-up period, 23% of patients developed locoregional recurrences, 40% of them with evidence of PnI or LVI, and 26% of those without peri-tumoral invasion (Figure 6).

Patients without perineural invasion (PnI−) demonstrated more favorable survival outcomes compared to those with perineural invasion (PnI+). The median overall survival for PnI− patients was 6.3 years (95% CI: 5.5–7.1), whereas it was markedly reduced in the PnI+ group at 3.0 years (95% CI: 1.8–6.0). At two and five years, overall survival rates in the PnI− cohort were 77% and 58%, respectively, compared to 58% and 41% in the PnI+ group. Similarly, cancer-specific mortality at two years was higher among PnI+ patients (23%) than PnI− patients (13%), with this trend persisting at five years (25% vs. 21%).

Locoregional and local recurrence rates were also elevated in the PnI+ cohort. At two years, locoregional recurrence occurred in 28% of PnI+ patients compared to 19% in PnI−, and by five years, these rates rose to 34% and 28%, respectively. Local recurrence was similarly increased among those with perineural invasion (28% at two years and 34% at five years) compared to those without (16% and 25%, respectively).

A similar pattern was observed in patients with lymphovascular invasion (LVI+). Median survival was shorter in LVI+ patients at 1.9 years (95% CI: 1.2–3.9), compared to 6.5 years (95% CI: 5.7–7.7) in LVI− individuals. Overall survival at two and five years was 50% and 24% in the LVI+ group, whereas the corresponding figures in LVI− patients were 78% and 61%. Cancer-specific mortality was markedly elevated in the LVI+ cohort, with 33% and 44% at two and five years, respectively, versus 12% and 18% in the LVI− group.

Furthermore, LVI+ patients experienced substantially higher rates of both locoregional and local recurrence. Locoregional recurrence was observed in 43% at two years and 46% at five years in the LVI+ group, in contrast to 17% and 27% among LVI− patients. Local recurrence followed a comparable trend, with rates of 41% and 44% in LVI+ patients versus 14% and 24% in those without lymphovascular invasion.

Survival estimates at 2 and 5-year survival and event probabilities stratified by the presence of PnI and LVI are presented in Table 2, while binominal multivariate regression models that predict the presence of perineural and lymphovascular invasion through other demographic and clinical features are given in Table 3.

### 3.3. Association of PnI and LVI with Initial Presence of Lymph Nodes Metastasis

In univariate analysis, tumor size was significantly associated with the presence of lymph node metastasis at the time of primary surgery, with increasing T-stage correlating with higher odds ratios (ORs). This remained significant even after adjustment. Using T1 tumors as reference, the adjusted OR and 95% CI to develop locoregional lymph nodes metastases for T2, T3, and T4 tumors were 2.13 (CI: 1.25–3.65), 3.09 (CI: 1.48–6.47) and 2.66 (95% CI: 1.42–5.00), respectively.

The effect of PnI was evident in the univariate and multivariate analysis with a crude OR of 2.24 (CI: 1.26–4.00) and an adjusted OR of 1.85 (1.00–3.43). LVI demonstrated a strong and consistent association in both univariate and multivariate analyses. In univariate analysis, LVI+ cases had an OR of 4.32 (CI: 2.41–7.99), and this remained significant after adjustment for other covariates in multivariate analysis (OR 2.72, CI: 1.45–5.24).

These results underscore the independent prognostic relevance of perineural and lymphovascular invasion in predicting the presence of lymph node metastases (Table 4).

### 3.4. Survival Analysis

Considering the endpoints Overall Survival (OS), Oral Cancer Specific Survival (OCSS), Locoregional Recurrence-Free Survival (LRRFS), Local Recurrence-Free Survival (LRFS), and Distant Metastasis-Free Survival (DMFS), increasing age was consistently associated with poorer overall survival (HR ~ 1.04, *p* < 0.001). A Charlson Comorbidity Index (CCI) score ≥ 1 was also significantly linked to worse OS (HR 1.54, *p* = 0.002). Gender showed a trend toward better OS for females, reaching significance for PnI patients (HR 0.75, *p* = 0.045), but not for LVI individuals (HR 0.80, *p* = 0.12).

Current smokers had a markedly worse OS in both PnI and LVI groups (HR 2.23 and 2.07, both *p* < 0.001), while former smokers showed a non-significant trend toward poorer OS. Excessive alcohol consumption was not significantly associated with any survival outcomes in either analysis.

UICC stage was a strong prognostic factor. Compared to stage I, stages III and IV were consistently associated with significantly worse OS, OCSS, and DMFS. For example, in the PnI group, stage IV was associated with OS (HR 1.98, *p* < 0.001) and DMFS (HR 5.49, *p* < 0.001); in the LVI group, stage IV similarly predicted poor outcomes across all endpoints (e.g., DMFS HR 4.37, *p* < 0.001).

In terms of tumor pathology, perineural invasion (PnI) was associated with worse OS (HR 1.50, *p* = 0.031) but did not significantly affect other outcomes, whereas lymphovascular invasion (LVI) emerged as a strong negative prognostic factor across all endpoints, including OS (HR 1.88, *p* < 0.001), OCSS (HR 2.19, *p* = 0.005), and DMFS (HR 2.38, *p* = 0.010).

In summary, age, comorbidity burden, smoking, advanced UICC stage, and pathological features such as LVI and PnI were key independent predictors of survival in oral cancer patients, with LVI showing broader prognostic impact than PnI (detailed results are shown in Table 5 and Table 6).

In patients with early-stage (Stage I–II) disease, PnI and LVI showed weak and uncertain associations with OS presented by small effect estimates and wide confidence intervals. The HR for OS for PnI was 1.20 (CI: 0.66–2.18) and for LVI was (HR 1.26, 95% CI: 0.52–3.07). However, the effect estimates for OCSS, LRRFS and LRFS were stronger ranging between 2.19 and 3.24, yet still imprecise. This is mainly due to the small sample size of patients presenting with evident PnI and LVI in patients with stage I–II disease.

Among patients with advanced disease (Stage III–IV), both PnI and LVI were significantly associated with worse OS: PnI HR 1.83 (CI: 1.15–2.93) and LVI HR 1.70 (CI: 1.15–2.51). For LVI, the strength of the effect estimate remained constant for OCSS, LRRFS and LRFS, despite the effect estimate being less precise that for OS. However, for PnI, the effect estimate observed for OS was nullified for other survival outcomes.

**Figure 7:**
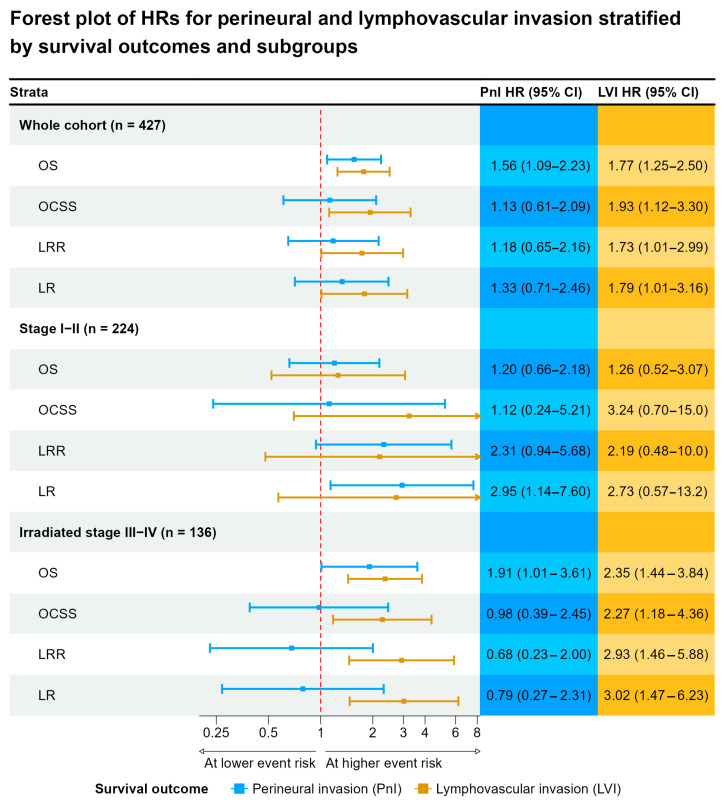
The forest plot presents hazard ratios (HRs) and 95% confidence intervals (CIs) for the impact of perineural invasion (PnI) and lymphovascular invasion (LVI) on various survival outcomes in patients with oral cancer, stratified by disease stage and treatment subgroup. It demonstrates that both perineural and lymphovascular invasion are important adverse prognostic indicators in oral cancer, but LVI appears to be the more consistent and potent predictor—especially in advanced-stage and irradiated patients. While PnI contributes to worse outcomes primarily through its impact on OS in the full and advanced cohorts, LVI shows broader associations across OS, OCSS, and recurrence outcomes, particularly in the irradiated advanced-stage subgroup.

In the subgroup of advanced-stage patients who received postoperative iradiotherapy, the effect estimates for PnI and LVI remained constant. Although, both PnI and LVI significantly predicted worse OS (PnI HR 1.91, CI: 1.01–3.61; LVI HR 2.35, CI: 1.44–3.84). Notably, LVI was a significant predictor of OCSS (HR 2.27, CI: 1.18–4.36), LRR (HR 2.93, CI: 1.46–5.88), and LR (HR 3.02, CI: 1.47–6.23), underscoring its particularly detrimental prognostic value in this subgroup. PnI did not show significant associations for these outcomes (Figure 7).

## 4. Discussion

### 4.1. PnI and LVI as Histologic Markers and Prognostic Risk Factors

The intra- and peritumoral presence and potential function of lymphatic and blood vessels in tumors remain controversial. Recently, lymphovascular invasion (LVI) has been postulated as a prognostic histologic marker for poor prognosis in OSCC [28]. Several reports showed that lymphoangiogenesis was closely related to lymphatic metastasis, providing additional conduits for disseminating cancer cells [29,30,31,32].

Similarly, the effects of perineural invasion (PnI) on local disease control and mortality have been explored in multiple studies and have been shown to predict both reduced survival and increased local recurrence independently [10,33]. Although relatively high rates of PnI are reported in early-stage disease, there is no consensus on whether patients with PnI require adjuvant therapy in the absence of additional adverse histopathologic findings [11,34].

In contrast to the present study, the vast majority of previous reports, including a reasonably high number of patients, retrieved the survival results from a national cancer registry and not from a follow-up-controlled institutional cancer database. Therefore, only the lymph node metastasis at the time of diagnosis and/or overall survival was set as an endpoint of the outcome [14,35]. However, especially for the PnI and LVI, locoregional control and, thus, recurrence-free survival are the crucial endpoints to investigate besides cancer-specific survival [35,36].

In a large series of patients (n = 1524), Nair et al. showed that PnI reduced disease-free and overall survival in T1–4 OSCC on multivariate analysis [10]. However, other studies have failed to show this effect [13,37]. For example, Chen et al. demonstrated in a recent study on 422 patients that PnI and LVI were not significant risk factors for locoregional control and overall survival for early-stage OSCC patients. Consequently, adjuvant radiotherapy could not provide an additional benefit for disease control and overall survival in patients with stage I/II with PnI and/or LVI [37].

Given the low prevalence of PnI and LVI, especially in the early stage of OSCC, it becomes clear that it is reasonably difficult to erase a high number of well-controlled patients in a reliable cohort in which a standardized histologic diagnosis over the years was carried out. Mostly, a reciprocal effect is evident: either there is a limited number of patients in a single-center cohort with high standardization and consistent diagnostic criteria or a population-based study with multi-centric, inhomogeneous data from a national health registry, where numerous pathologists with incoherent criteria for the definition of PnI and LVI are involved.

Furthermore, several factors may complicate the assessment of the impact of PnI and LVI on local tumor recurrence. One of them is the interaction between tumor size-related adjuvant radiotherapy and the presence of peri-tumoral invasion (PnI, LI, or VI). The other one is that certain survival events (such as death from causes other than oral cancer) may mask the occurrence of a local recurrence. These competing risks and cofounders require a careful selection and application of specific statistical evaluation methods for survival analysis.

To address these questions, we examined the individual role of peritumoral invasion on the survival outcomes in a prospective cohort of patients with non-metastatic OSCC. While stratifying the entity of this invasion into the categories PnI and LVI, the hazard ratio was adjusted for the competing risk factors, T-status, and cervical lymph node involvement (N+).

In a recent population-based study including 745 patients, lymphatic invasion (LI) alone was postulated as an independent risk factor for poor OS and DFS [38]. This effect, however, was not evident in a previous study by Adel et al. including 571 individuals [18]. This discrepancy may be attributed to the interpretation of the histologic findings over the long years of the cohort (up- or downgrade the feature LI or VI), or to the role of the cofounders, such as tumor size and stage, in the interpretation of results and related analysis.

Therefore, we investigated the correlation of tumor size as well as PnI and LVI with the presence of the most relevant and established prognostic risk factors, namely the initial presence of regional lymph node metastasis in a uni- and multivariate analysis. The results encountered here confirms that, besides tumor size, LVI, but not PnI represents an independent risk factor for lymph node involvement at the time of diagnosis, which in turn aggravates clinical stage and contributes to the poor prognosis.

Poleksic and Kalwaic first suggested that the tumor’s penetration of the vascular channels is an indicator of the tumor’s aggressive character in head and neck SCC [39]. Two decades ago, the role of vascularity in many tumor entities was investigated, and the new concept of tumor angiogenesis was introduced and established. However, higher microvascular density, which was found to rise with increasing T and N stages, did not correlate with VEGF expression or survival in patients with OSCC [40,41,42]. Interestingly, lymphovascular invasion (LVI), as investigated in the present study shows a significant correlation with poor OS, OCSS and LRR.

In contrast, Adel et al. investigated the lymphatic and vascular invasion independently in OSCC and assessed their correlation with overall survival. They found that neither lymphatic nor vascular invasion impacts locoregional recurrence or distant metastasis after treatment. However, since lymphatic and vascular invasion were found to be strongly associated with various pathological factors and higher stage, most OSCC patients with LI or VI received postoperative radiotherapy or chemoradiotherapy. In other words, only two cases of OSCC patients with lymphatic invasion and 3 cases with vascular invasion did not receive any postoperative treatment. Due to these limited patient numbers, the survival outcome could be biased [18]. One of the strengths of the present study is that a considerably high proportion of included patients had peritumoral invasion but did not receive adjuvant radiotherapy. This reduces possible bias and enables estimating the prognostic effect of such invasion in a reliable regression model.

### 4.2. Effect of Postoperative Radio(Chemo)Therapy on Survival in Patients with PnI and LVI

Multiple trials have shown that postoperative radiotherapy (PORT) enhances local control and DFS in patients with PnI, especially in early-stage, node-negative OSCC. According to meta-analyses and retrospective investigations, PORT is linked to better DFS rates than surgery alone in stage I/II patients with PnI [43,44].

While adjuvant radiotherapy has been shown to decrease the hazard of recurrence and significantly improve locoregional control, the benefit for overall survival is less evident, though, as some studies—with a comparable number of patients to the present one—show no discernible difference in OS between PORT and non-PORT groups, particularly in low-risk patients or in the early stages of the disease [45].

The same effect was observed in the present study in I/II-stage PnI patients who did not receive radiotherapy initially. The incidence of local recurrence was higher, but OS remained unimpaired. This may be attributed to the subsequent therapy after recurrence which usually includes radio (chemo) therapy in the recommended guidelines, thus shifting patients onto the subgroup with higher stage and initial PORT, those who showed a benefit form PORT in view of all survival outcomes (OS, OCSS, LRR, LR).

While radiotherapy attenuated PnI effect on OS in irradiated stage III/IV patients, all other estimated survival endpoints (OCSS, LRR, and LR) remained significantly poor despite the adjuvant therapy in the LVI subgroup. This underscores the crucial role of LVI in clinical disease course, means that those patients had a higher risk of developing metastasis. These patients may benefit form a strict follow-up in which systemic detection of metastasis could be provided (e.g., whole-body PET-CT) along with a lower threshold for adjuvant systemic treatment escalation initially.

### 4.3. Strengths and Limitations of the Study

One of the study’s strengths is the single center setting, which ensures standardized documentation, uniform recording of clinical data, and, above all, a uniform definition and interpretation of the histological variables PnI and LVI. All histologic sections were re-evaluated by two pathologists who were blinded to patients’ data and assessed according to the current international standard. This is especially crucial concerning the definition of PnI, as this has undergone several revisions since the first description. The most recent redefinition representing the current accepted standard was introduced by Liebig et al. in 2009 [25]. Since most studies acquired old data from the pathology reports and/or national cancer registries were adopted as such and not reassessed, a wide range of incidence of PnI in OSCC patients has been reported and lead to controverse results.

The same is true for the definition of LI, VI or both (as LVI). Although LVI is roughly defined as the presence of tumor cells within definite endothelial-lined spaces, either lymphatic or blood vessels, it is subject to a wide scope of interpretation in view of the presence of malignant cells within an endothelial-lined space or if they are focally adherent to the vessel wall [14]. The recent development in the immunohistological detection of such an invasion has aggravated this aspect and emphasized the importance of independent reevaluation of histologic sections within cohort studies.

To reduce false-negative findings due to sampling limitations, multiple sections of each tumor were histologically examined, including areas from the main tumor as well as the interface with adjacent tissue. Although all tissue blocks were evaluated, serial sections of individual blocks were not prepared, and additional immunohistochemical or special staining (e.g., Elastica van Gieson for VI, Podoplanin for LI and S100 for PnI) was not routinely performed. We deliberately handled it this way to replicate the realistic clinical situation, as this is not a common practice in routine diagnostics either. Therefore, small or focal areas of peritumoral invasion might have been missed, which is also a limitation of this study.

Another limitation of the present study lies in its exclusive focus on histomorphological features characterizing tumor growth within and along anatomical structures. The analysis did not account for the immune response or growth patterns at the invasive front, both of which have increasingly been recognized as relevant prognostic factors.

The present investigation, with its single-center, well-controlled character, brings to light a clinically relevant aspect but also shows some limitations. While the histopathology material was reviewed to ensure homogeneity of coherence with histopathologic criteria, the sample size and the inhomogeneous distribution of the patients who received various treatments (surgery only, surgery + radiotherapy) was limited. Additionally, changes in treatment protocols over the course of the prolonged patient inclusion period could also have influenced the survival outcomes. Further, patients with stage I/II disease with both PnI and LVI were underrepresented within the cohort; hence, a reliable statement in this subgroup remains questionable. Our data and conclusions should be validated in other prospective cohorts of patients with OSCC in the future.

## 5. Conclusions

Perineural invasion (PnI) and lymphovascular invasion (LVI) are emerging as potential descriptors for staging in the current TNM classification and in guiding therapy recommendations. Although their roles remain controversial, growing evidence supports their impact on survival outcomes.

This study demonstrates that both PnI and LVI are associated with poor prognosis, with LVI serving as a strong predictor of poor overall and cancer-specific survival, and recurrence. Future updates to the TNM staging system should incorporate the LVI status in the treatment guidelines for patients with OSCC toward encouraging treatment escalation, such as wider clear resection margin, postoperative chemoradiation, interstitial brachytherapy, and/or immune therapy. Furthermore, clinical studies are warranted to clarify the therapeutic benefit of this escalation, considering locoregional control, survival and quality of life within a prospective controlled trial.

## Figures and Tables

**Table 1:** Clinicopathological characteristics of 439 patients with oral squamous cell carcinoma (OSCC), stratified by the evidence of perineural invasion (PnI) and lymphovascular invasion (LVI). Percentages were calculated by column.

	Stratified by PnI		Stratified by LVI		Overall N = 439 (100%) ^1^
Variable	PnI− N = 385 (88%) ^1^	PnI+ N = 54 (12%) ^1^	LVI− N = 384 (87%) ^1^	LVI+ N = 55 (13%) ^1^	
Age	62 (54–71)	64 (51–72)	63 (54–72)	61 (53–67)	62 (54–71)
Gender					
Male	236 (61%)	28 (52%)	222 (58%)	42 (76%)	264 (60%)
Female	149 (39%)	26 (48%)	162 (42%)	13 (24%)	175 (40%)
CCI score					
0	261 (68%)	33 (61%)	256 (67%)	38 (69%)	294 (67%)
1≤	124 (32%)	21 (39%)	128 (33%)	17 (31%)	145 (33%)
Smoking					
Never	82 (22%)	11 (20%)	89 (24%)	4 (7.3%)	93 (22%)
Former or current	291 (78%)	43 (80%)	283 (76%)	51 (93%)	334 (78%)
Missing	12	0	12	0	12
Alcohol consumption					
None or moderate	164 (45%)	15 (29%)	163 (45%)	16 (31%)	179 (43%)
Excessive	198 (55%)	37 (71%)	199 (55%)	36 (69%)	235 (57%)
Missing	23	2	22	3	25
Tumor subsite					
Floor of mouth	131 (34%)	18 (33%)	123 (32%)	26 (47%)	149 (34%)
Anterior tongue	104 (27%)	20 (37%)	112 (29%)	12 (22%)	124 (28%)
Gum	84 (22%)	8 (15%)	77 (20%)	15 (27%)	92 (21%)
Cheek, Vestibule, retromolar	34 (8.8%)	6 (11%)	39 (10%)	1 (1.8%)	40 (9.1%)
Palate	32 (8.3%)	2 (3.7%)	33 (8.6%)	1 (1.8%)	34 (7.7%)
Tumor size					
T1	163 (42%)	12 (22%)	171 (45%)	4 (7.3%)	175 (40%)
T2	121 (31%)	22 (41%)	123 (32%)	20 (36%)	143 (33%)
T3	40 (10%)	7 (13%)	36 (9.4%)	11 (20%)	47 (11%)
T4	61 (16%)	13 (24%)	54 (14%)	20 (36%)	74 (17%)
Nodal disease					
N0	267 (69%)	27 (50%)	275 (72%)	19 (35%)	294 (67%)
N1	51 (13%)	8 (15%)	50 (13%)	9 (16%)	59 (13%)
N2a/b	45 (12%)	9 (17%)	41 (11%)	13 (24%)	54 (12%)
N2c/3	22 (5.7%)	10 (19%)	18 (4.7%)	14 (25%)	32 (7.3%)
UICC stage					
I	147 (38%)	7 (13%)	153 (40%)	1 (1.8%)	154 (35%)
II	64 (17%)	13 (24%)	70 (18%)	7 (13%)	77 (18%)
III	65 (17%)	9 (17%)	64 (17%)	10 (18%)	74 (17%)
IV	109 (28%)	25 (46%)	97 (25%)	37 (67%)	134 (31%)
Grade of differentiation					
Well	36 (9.4%)	2 (3.7%)	38 (9.9%)	0 (0%)	38 (8.7%)
Moderate	276 (72%)	39 (72%)	281 (73%)	34 (62%)	315 (72%)
Poor	72 (19%)	13 (24%)	64 (17%)	21 (38%)	85 (19%)
Missing	1	0	1	0	1
Resection margins					
R0	152 (39%)	13 (24%)	157 (41%)	8 (15%)	165 (38%)
R0cm	122 (32%)	22 (41%)	125 (33%)	19 (35%)	144 (33%)
R0hr	111 (29%)	19 (35%)	102 (27%)	28 (51%)	130 (30%)

^1^ Median (Q1–Q3); n (%).

**Table 2:** Survival estimates for OS, OCSS, LRR and LR at 2 and 5 years after surgery stratified by perineural and lymphovascular invasion.

	Overall Survival	Death from Oral Cancer	Locoregional Recurrence	Local Recurrence
**Variable**	At 2 Years			
Overall	75% (71–79%)	14% (11–18%)	20% (16–24%)	18% (14–22%)
Perineural invasion				
PnI−	77% (73–81%)	13% (10–17%)	19% (15–23%)	16% (12–20%)
PnI+	58% (46–73%)	23% (13–35%)	28% (15–41%)	28% (15–41%)
Lymphovascular invasion				
LVI−	78% (74–83%)	12% (8.6–15%)	17% (13–21%)	14% (11–18%)
LVI+	50% (38–65%)	33% (21–46%)	43% (28–57%)	41% (26–55%)
	At 5 years			
Overall	56% (51–61%)	21% (17–25%)	29% (24–34%)	26% (22–31%)
Perineural invasion				
PnI−	58% (53–64%)	21% (16–25%)	28% (23–34%)	25% (21–31%)
PnI+	41% (29–57%)	25% (14–38%)	34% (20–48%)	34% (20–48%)
Lymphovascular invasion				
LVI−	61% (56–66%)	18% (14–22%)	27% (22–32%)	24% (19–29%)
LVI+	24% (15–40%)	44% (30–57%)	46% (30–61%)	44% (28–58%)

**Table 3:** Results of two binominal multivariate regression models that predicts the presence of perineural and lymphovascular invasion through other demographic and clinical features. Significant results are highlighted in bold. Interestingly, PnI was strongly associated with being female, having relevant comorbidities and with anterior tongue cancers. LVI on the other hand was strongly correlated with previous and current smoking as well as poor differentiation. As expected, as the UICC stage increased so was the presence of PnI and LVI.

	Perineural Invasion (PnI)			Lymphovascular Invasion (LVI)		
Variable	OR	95% CI	*p*-Value	OR	95% CI	*p*-Value
Age	0.98	0.95–1.01	0.2	1.00	0.96–1.03	0.8
Gender						
Male	—	—		—	—	
Female	**2.02**	**1.08–3.81**	**0.028**	0.69	0.32–1.41	0.3
CCI score						
0	—	—		—	—	
1≤	**1.81**	**0.93–3.53**	**0.080**	1.12	0.54–2.27	0.8
Smoking						
Never	—	—		—	—	
Former	1.14	0.40–3.22	0.8	2.93	0.81–12.4	0.12
Current	0.91	0.37–2.34	0.8	**3.52**	**1.10–14.0**	**0.049**
Tumor subsite						
Floor of mouth	—	—		—	—	
Anterior tongue	**2.02**	**0.92–4.52**	**0.082**	1.13	0.48–2.64	0.8
Gum	0.65	0.23–1.68	0.4	1.21	0.50–2.90	0.7
Cheek, Vestibule, retromolar	1.69	0.54–4.85	0.3	0.18	0.01–1.01	0.11
Palate	0.50	0.07–1.97	0.4	0.21	0.01–1.20	0.2
UICC stage						
I	—	—		—	—	
II	**4.71**	**1.77–13.6**	**0.003**	**12.2**	**2.04–232**	**0.022**
III	**3.39**	**1.16–10.4**	**0.026**	**18.5**	**3.31–347**	**0.006**
IV	**7.71**	**3.13–21.4**	**<0.001**	**47.3**	**9.60–858**	**<0.001**
Grade of differentiation						
Well/Moderate	—	—		—	—	
Poor	1.19	0.56–2.37	0.6	**2.82**	**1.40–5.68**	**0.003**

Abbreviations: CI = Confidence Interval, OR = Odds Ratio.

**Table 4:** Results of a univariate and a multivariate regression model that predicts the presence of lymph node metastasis. Significant results are highlighted in bold.

	Univariate			Multivariate		
Variable	OR	95% CI	*p*-Value	OR	95% CI	*p*-Value
Gender						
Male	—	—		—	—	
Female	0.68	0.45–1.02	0.066	0.80	0.51–1.27	0.4
Tumor subsite						
Floor of mouth	—	—				
Anterior tongue	0.84	0.50–1.38	0.5			
Gum	1.29	0.76–2.20	0.3			
Cheek, Vestibule, retromolar	0.59	0.26–1.26	0.2			
Palate	0.73	0.31–1.61	0.5			
Smoking						
Never	—	—		—	—	
Former	1.51	0.77–2.96	0.2	1.20	0.58–2.48	0.6
Current	1.58	0.95–2.70	0.086	1.13	0.64–2.01	0.7
Tumor size						
T1	—	—		—	—	
T2	**2.91**	**1.77–4.84**	**<0.001**	**2.13**	**1.25–3.65**	**0.005**
T3	**4.71**	**2.39–9.43**	**<0.001**	**3.09**	**1.48–6.47**	**0.003**
T4	**3.72**	**2.07–6.76**	**<0.001**	**2.66**	**1.42–5.00**	**0.002**
Grade of differentiation						
Well/Moderate	—	—		—	—	
Poor	**1.80**	**1.11–2.91**	**0.017**	1.45	0.85–2.47	0.2
Perineural invasion						
None	—	—		—	—	
Evident	**2.24**	**1.26–4.00**	**0.006**	1.85	1.00–3.43	0.051
Lymphovascular invasion						
None	—	—		—	—	
Evident	**4.32**	**2.41–7.99**	**<0.001**	**2.72**	**1.45–5.24**	**0.002**

Abbreviations: CI = Confidence Interval, OR = Odds Ratio.

**Table 5:** Hazards ratios (HR) estimates with their 95% confidence intervals from four Cox regression models for perineural invasion for five different survival outcomes. Significant results are highlighted in bold. Perineural invasion was associated with worse overall survival. However, the magnitude of effect was weaker for other survival outcomes.

	OS			OCSS			LRRFS			LRFS			DMFS		
Variable	HR	95% CI	*p*-Value	HR	95% CI	*p*-Value	HR	95% CI	*p*-Value	HR	95% CI	*p*-Value	HR	95% CI	*p*-Value
Age	1.04	1.02–1.05	**<0.001**												
CCI score															
0	—	—													
1≤	1.54	1.17–2.03	**0.002**												
Gender															
Male	—	—		—	—		—	—		—	—		—	—	
Female	0.75	0.56–0.99	**0.045**	0.92	0.54–1.55	0.8	1.01	0.66–1.55	>0.9	0.98	0.62–1.54	>0.9	0.82	0.42–1.62	0.6
Smoking															
Never	—	—		—	—		—	—		—	—		—	—	
Former	1.61	1.00–2.59	**0.049**	1.47	0.65–3.33	0.4	1.06	0.55–2.03	0.9	1.06	0.53–2.09	0.9	1.31	0.48–3.54	0.6
Current	2.23	1.49–3.34	**<0.001**	1.49	0.76–2.93	0.3	0.94	0.56–1.60	0.8	0.90	0.52–1.57	0.7	1.35	0.59–3.14	0.5
Alcohol consumption															
None or moderate	—	—		—	—		—	—		—	—		—	—	
Excessive	1.08	0.80–1.45	0.6	1.03	0.61–1.74	>0.9	0.87	0.55–1.39	0.6	0.89	0.55–1.44	0.6	1.01	0.50–2.03	>0.9
UICC stage															
I	—	—		—	—		—	—		—	—		—	—	
II	1.00	0.67–1.49	>0.9	1.62	0.67–3.90	0.3	0.98	0.49–1.95	>0.9	1.04	0.50–2.13	>0.9	0.98	0.30–3.23	>0.9
III	1.89	1.30–2.77	**<0.001**	3.77	1.78–7.99	**<0.001**	1.69	0.90–3.18	0.10	1.57	0.80–3.07	0.2	3.22	1.27–8.16	**0.014**
IV	1.98	1.42–2.76	**<0.001**	4.65	2.37–9.10	**<0.001**	2.74	1.67–4.50	**<0.001**	2.73	1.62–4.61	**<0.001**	5.49	2.47–12.2	**<0.001**
Perineural invasion															
PnI-	—	—		—	—		—	—		—	—		—	—	
PnI+	1.50	1.04–2.17	**0.031**	1.05	0.55–2.00	0.9	1.22	0.67–2.24	0.5	1.34	0.73–2.48	0.4	1.02	0.46–2.30	>0.9

Abbreviations: CI = Confidence Interval, HR = Hazard Ratio, OS = Overall survival, OCSS = Oral cancer specific survival, LRRFS = Locoregional recurrence free survival, LRFS = Local recurrence free survival, DMFS = Distant metastasis free survival.

**Table 6:** This shows the hazards ratios (HR) estimates with their 95% confidence intervals from four Cox regression models for lymphovascular invasion for five different survival outcomes. Lymphovascular invasion was associated with worse survival regardless of the examined outcome. Interestingly, the HR for distant metastasis was also notably very high.

	OS			OCSS			LRRFS			LRFS			DMFS		
Variable	HR	95% CI	*p*-Value	HR	95% CI	*p*-Value	HR	95% CI	*p*-Value	HR	95% CI	*p*-Value	HR	95% CI	*p*-Value
Age	1.04	1.02–1.05	**<0.001**												
CCI score															
0	—	—													
1≤	1.54	1.17–2.02	**0.002**												
Gender															
Male	—	—		—	—		—	—		—	—		—	—	
Female	0.80	0.60–1.06	0.12	1.00	0.59–1.72	>0.9	1.05	0.68–1.61	0.8	0.99	0.63–1.57	>0.9	0.87	0.44–1.72	0.7
Smoking															
Never	—	—		—	—		—	—		—	—		—	—	
Former	1.54	0.96–2.47	0.074	1.35	0.61–3.02	0.5	0.98	0.51–1.88	>0.9	1.03	0.52–2.04	>0.9	1.22	0.45–3.27	0.7
Current	2.07	1.38–3.10	**<0.001**	1.31	0.68–2.55	0.4	0.87	0.52–1.47	0.6	0.87	0.50–1.52	0.6	1.22	0.53–2.80	0.6
Alcohol consumption															
None or moderate	—	—		—	—		—	—		—	—		—	—	
Excessive	1.14	0.85–1.53	0.4	1.06	0.64–1.76	0.8	0.87	0.55–1.38	0.6	0.90	0.56–1.44	0.7	0.98	0.50–1.96	>0.9
UICC stage															
I	—	—		—	—		—	—		—	—		—	—	
II	1.01	0.68–1.50	>0.9	1.53	0.64–3.63	0.3	0.96	0.48–1.92	>0.9	1.02	0.49–2.12	>0.9	0.92	0.28–2.96	0.9
III	1.69	1.15–2.49	**0.008**	3.35	1.57–7.13	**0.002**	1.56	0.81–2.98	0.2	1.57	0.78–3.14	0.2	2.75	1.06–7.18	**0.038**
IV	1.85	1.32–2.59	**<0.001**	3.68	1.83–7.39	**<0.001**	2.41	1.44–4.03	**<0.001**	2.71	1.57–4.67	**<0.001**	4.37	1.92–9.94	**<0.001**
Lymphovascular invasion															
LVI-	—	—		—	—		—	—		—	—		—	—	
LVI+	1.88	1.32–2.69	**<0.001**	2.19	1.27–3.78	**0.005**	1.88	1.08–3.26	**0.024**	1.92	1.08–3.42	**0.026**	2.38	1.23–4.58	**0.010**

Abbreviations: CI = Confidence Interval, HR = Hazard Ratio, OS = Overall survival, OCSS = Oral cancer specific survival, LRRFS = Locoregional recurrence free survival, LRFS = Local recurrence free survival, DMFS = Distant metastasis free survival.

## Data Availability

The data supporting the findings of this study are not publicly available due to privacy and ethical restrictions.
